# Engraved Microwave Metasurfaces for Potential Application in Honey Quality Control

**DOI:** 10.1021/acsomega.5c04380

**Published:** 2025-10-01

**Authors:** Argyri Drymiskianaki, Klytaimnistra Katsara, Vassilis M. Papadakis, Zacharias Viskadourakis, George Kenanakis

**Affiliations:** † Department of Materials Science and Engineering, 162235 University of Crete, Heraklion, Crete GR-70013, Greece; ‡ Institute of Electronic Structure and Laser (IESL), Foundation for Research and Technology − Hellas (FORTH), N. Plastira 100, Vassilika Vouton, Heraklion GR-70013, Greece; § Department of Agriculture, 112178 Hellenic Mediterranean University, Estavromenos, Heraklion, Crete GR-71410, Greece; ∥ Department of Industrial Design and Production Engineering, University of West Attica, Athens GR-12243, Greece

## Abstract

In this study, millimeter-scale metasurfaces are examined concerning their capability as potential honey quality control sensors. In particular, complementary split-ring resonator metasurfaces were developed through the Computer Numerical Control engraving method. Under unloaded conditions, the metasurfaces exhibited fundamental resonance frequencies in the range of 3–6 GHz, depending on their size as well as the measurement orientation. Moreover, their electromagnetic response was studied in the presence of different honey types, such as chestnut, pine, and orange blossom honey. A corresponding resonance frequency shift was observed, suggesting the distinct electromagnetic response of the fabricated structures with respect to the honey type. Furthermore, the hereby studied resonators were tested against various types of honey adulteration, such as sweeteners (sugar and maple syrup), as well as microplastic contamination. The metasurfaces exhibit a discrete resonance frequency shift along with modulation of the resonance intensity in honey adulteration, enabling them to act as efficient honey quality detectors. In addition, their electromagnetic performance was compared to other state-of-the-art spectroscopic techniques dedicated to honey contamination, in particular, Raman and FTIR. It was found that spectroscopic evidence is consistent with the metasurface electromagnetic response, giving credence to their performance. All in all, it is evident that the hereby studied metasurfaces exhibit significant performance in honey adulteration sensing, allowing their potential application as sensors for honey quality control.

## Graphical abstract

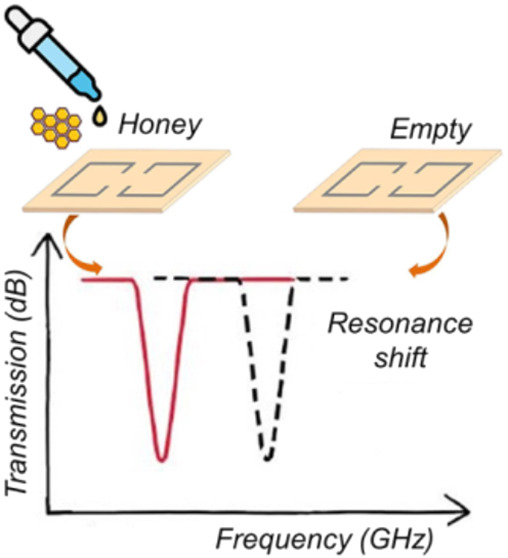

## 1 Introduction

Honey, the oldest known sweetener, has a rich history of use and production dating back thousands of years.^1^ It is produced by bees from carbohydrate-containing exudates of plants (blossom or nectar kinds of honey) or excretions of plant-sucking insects on living parts of plants.^2^ It primarily consists of sugars (fructose, glucose, sucrose, and oligosaccharides), enzymes, vitamins, minerals, organic acids, essential oils, esters, proteins, polyphenols, pigments, beeswax, and pollen, which contribute to its color, smell, and flavor.^3^

Nowadays, honey production and distribution are a valuable income source for many countries around the world.^4,5^ With the annual honey demand continuously increasing, the global honey trade market is continuously growing.^6^ In order to reduce costs, honey companies mix honey with sugar-based sweeteners, increasing the overall honey volume and degrading nutrition quality. Even worse, beekeepers increase their production by feeding bees with sweeteners, reducing the quality of the produced honey.^3^ Hence, as honey adulteration becomes a growing concern for individuals’ feeding habits, novel methods and procedures regarding honey quality monitoring are highly required.

Up to date, honey adulteration is realized using state-of-the-art spectroscopic and analytical techniques such as Raman and Fourier-transform infrared spectroscopy (FTIR),^7,8^ gas chromatography–mass spectrometry (GC–MS),^9^ high-performance liquid chromatography (HPLC),^10^ and liquid chromatography with tandem mass spectrometry (LC-MS/MS)^11^ methods. Fluorescence spectroscopy,^12^ photoacoustic spectroscopy,^13^ and X-ray diffraction^14^ are also used for honey authentication and adulteration studies. Furthermore, Raman, FTIR, DLLME–GC–MS, and electrospray Ionization mass spectrometry (ESI-MS) techniques have been used in the past few years for the detection and identification of microplastics in honey.^15–19^ The aforementioned techniques are well-established, accurate, and high-resolution procedures. Nevertheless, all of them are laboratory-oriented and time-consuming techniques. Moreover, the corresponding infrastructure is expensive and complicated in its use, while well-trained personnel might be required, and it is often accompanied by high maintenance costs.

Based on that, the prospect of developing highly sensitive and fast-responsive sensors, which are also cheap, can be used remotely, are portable, and easy to use, remains a challenging task. Within this framework, metasurfaces could be quite interesting candidates. Metasurfaces (MSs) are artificially made, planar structures, exhibiting electromagnetic properties that are not normally met in natural materials, such as perfect lensing, maximum electromagnetic absorption, cloaking capability, high-frequency magnetism, dynamic modulation of Terahertz (THz) radiation, reverse Doppler effect, etc.^20^ A MS consists of multiple structural elements of a specific geometry called meta-atoms, which are periodically arranged in space.^21^ In its unitary form, MS can entail a single meta-atom (MA), without sacrificing its electromagnetic performance. The electromagnetic response of the single meta-atom MS can be tuned by tailoring the meta-atom’s geometrical characteristics. This reconfigurability option turns MSs into ideal candidates for various electromagnetic applications, including energy harvesters, miniaturized antennas, electromagnetic shields, as well as sensors.^22^

Among other MS topologies, split-ring resonators (SRRs) are resonant elements formed by a ring-shaped conductive pattern including an opening gap. In order to understand the resonance behavior of such an MS, each SRR can be considered as an RLC circuit where the conductive parts of the meta-atom correspond to the resistive and the inductive parts, whereas the gap represents the capacitance. Each RLC circuit resonates at a certain frequency, given by the formula *f*_resonance_ ∝ 1/2π√*LC*, where *L* is the effective inductance of the SRR, and the capacitance factor is inversely proportional to the material’s dielectric permittivity. As a consequence, the resonance frequency is intrinsically linked to the dielectric properties of the surrounding medium. The SRR topology enables the ability to confine electromagnetic fields in the adjacent conductive paths of the structure, with the highest field intensity being localized in the gap of the structure.^23^ Hence, the placement of a dielectric material into the gap of the SRR alters the effective capacitance (through the interaction with the localized electric field) and, consequently, its resonance frequency,^22^ enabling its sensing capability. Notably, recent reports exist regarding the use of SRRs as sensors with respect to solids, liquids, and gases.^24,25^ The negative counterpart of an SRR is called a complementary split-ring resonator (CSRR) and possesses an EM behavior identical to the SRR, described by the Babinet principle.^26^ CSRRs have also been gaining ground as sensing devices, mainly due to the increased active sensing zone they possess compared to the typical SRRs, where the detection area is limited in the narrow gap region.^27,28^ Although physically plane, the impact of the sample volume on the electromagnetic response of the resonator also implies a volumetric contribution to the sensing mechanism.^29^ The interaction between the localized electric field and the material under test can define a volumetric sensing region, confined within the CSRR-engraved path, which is usually in the order of several mm^3^ depending on the selected topology. Until now, SRR and CSRR sensors have been successfully employed in several studies regarding food quality control.^30–32^

So far, there have been a considerable number of studies regarding the sensing capabilities of resonating elements in honey quality control. Nonetheless, in the vast majority, either conventional antennas or SRRs are investigated;^33–35^ a few reports explore the use of CSRRs as honey sensors.^36^ Those that do, either focus primarily on their qualitative electromagnetic response or simultaneously exploit the synergetic action of spectroscopic methods to corroborate the sensor effectiveness.^36^ To our knowledge, there are still no studies exploring the implementation of CSRRs against honey adulteration as stand-alone sensors.^37^

In view of all of the above, the present study explores the sensing ability of rectangular CSRRs, developed via the Computer Numerical Control (CNC) engraving technique. Apart from the hitherto underexplored CSRR topology for the assessment of honey quality levels, the novelty of the proposed sensor also lies in the subtractive design of the CSRRs. The sensing material is placed directly into the engraved area of the sensor. This eliminates the need for an external capillary network or a separate microfluidic channel, further miniaturizing the system. Moreover, CNC engraving technique is considered as an additive (subtractive) manufacturing procedure, which exhibits beneficial advantages against conventional SRR manufacturing processes, such as printed circuit board (PCB),^38^ inkjet printing,^39^ thermal evaporation, lithography, etc., as it is easy to use, of low cost, and environmentally friendly. This fact enables the possibility for large-scale production, with a concurrent decrease in fabrication time and production expenses. Hence, a sensing approach that utilizes CSRRs grown using CNC technology raises an intriguing field to explore.

In this context, CSRRs with varying dimensions were fabricated and comprehensively characterized regarding their resonant behavior in the microwave regime. All grown CSRRs show relatively sharp dips in their transmission spectra, indicative of resonance, in the regime of 3–6 GHz, depending on their dimensions. The sensing performance of the MSs was examined under different varieties of honey. The MSs were found to be highly responsive to different honey types, exhibiting nondegenerate resonance peaks for each honey variety. Furthermore, their discriminative ability among commonly used honey adulterants, such as sweeteners, was also tested. Several honey solutions, including commonly used sweeteners (sugar, maple syrup), were prepared. In that case, CSRRs respond adequately to the presence of different honey solutions, enabling their detection capabilities in honey adulteration.

Moreover, honey contamination from plastic microparticles was also examined. Aqueous solutions including honey and poly­(ethylene terephthalate) (PET) microparticles were prepared. The MSs exhibited good sensing ability also in the contamination scenario, demonstrating discrete resonance peaks in the presence of PET. The EM response of the MSs in the above-examined cases was cross-examined with corresponding findings obtained from Raman, FTIR, and dielectric permittivity experiments. Interestingly, a comparative analysis revealed significant consistency in the key parameters assessed by each method, indicating a clear correlation between them. Given that the above spectroscopic techniques belong among the most indicated procedures regarding the quality assessment of edibles in the food industry, it can be safely presumed that CSRRs hold great potential to function effectively as honey quality control sensors. This is a promising approach for low-cost and easy quality control of consumable honey.

## 2 Experimental Section

### 2.1 Metasurface Fabrication

Rectangular CSRR single meta-atom metasurfaces were chosen for the purpose of the current study. Notably, rectangular SRRs have been extensively studied in the past.^40^ Even more, CSRRs have been studied as potential water as well as oil adulteration sensors.^29,41^ The mechanical engraving method was used in order to develop such CSRRs, using a home-built CNC router. Corresponding CSRR drawings were developed using the open-source drawing software EASEL (Inventables Inc., Chicago), which is also dedicated to appropriately transforming each drawing to corresponding g-code files that can be read by the CNC router. Then, the router moves in all three directions upon engraving, by using a thin metallic carpenter blade (diameter: Ø 1 mm), so that the final engraved subject is fabricated. The substrate material used for engraving was a typical plain FR-4 surface (1 mm thickness), covered by a 35 μm-thick film of pure copper (Cu). Through the engraving procedure, the blade removed certain areas of the Cu film so that complementary CSRRs were grown (Figure 1a,b; sample dimensions can be found in Table 1). Each CSRR entails two engraved rings with opposing gaps, intensifying their resonant response through EM coupling; the strength of the latter is dependent on factors such as the planar orientation of the rings as well as their proximity given by distance D (Figure 1b).^42^ By taking into account the corresponding analytical models for the studied CSRRs (Figures S3 and S4), the highest electric field intensity is found in the CSRR gap; thus, a mirrored configuration is favored in order to achieve a stronger interaction between the two opposing fields, enhancing the overall coupling strength. In all cases, the depth of the engraved areas was kept at 0.2 mm.

**Figure 1:**
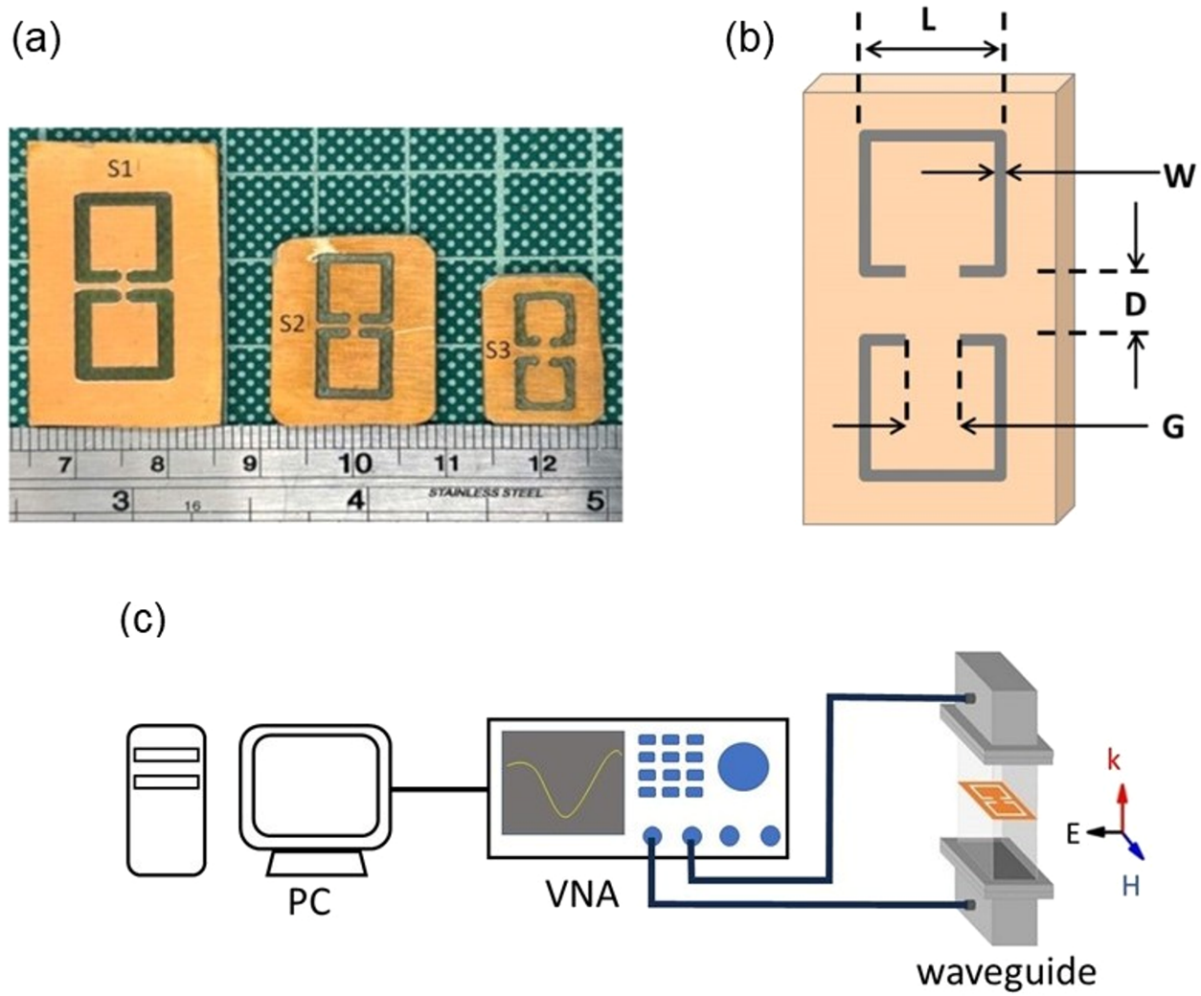
(a) General view of the engraved CSRRs used in this study. (b) Schematic illustration of the selected CSRR topology with the critical dimensions denoted. (c) Experimental setup drawing for EM measurements.

**Table 1:** Code Names and Dimensions and Engraved Area Volume, for all CSRRs

**CSRR**	***L*** (mm)	***W*** (mm)	***G*** (mm)	***D*** (mm)	***V*** (mm^**3**^ **)**
**S1**	10.3 ± 0.1	1.5 ± 0.1	1.1 ± 0.1	0.8 ± 0.1	10 ± 2
**S2**	7.9 ± 0.1	1.3 ± 0.1	1.3 ± 0.1	0.5 ± 0.1	7 ± 1
**S3**	6.5 ± 0.1	1.1 ± 0.1	1.3 ± 0.1	1.2 ± 0.1	4 ± 1

### 2.2 Metasurface Electromagnetic Characterization

All of the fabricated CSRRs were characterized by their electromagnetic behavior. More specifically, transmission spectra were collected, using a combination of a P9372A Vector Network Analyzer (VNA) (Keysight, California) and WR284 (2.4–4.8 GHz), WR187 (3.5–7 GHz), and WR137 (5–10 GHz) waveguides (corresponding experimental setup is shown in Figure 1c) so that a frequency range of 2.4–9 GHz is covered. Details regarding the setup and the measurement procedure were previously described.^29,40,41^ Two different configurations were investigated, i.e., the CSRR being oriented in a way that the electric field component is parallel (TE orientation, drawing in Figure 2a) and perpendicular to the gap (TM orientation, drawing in Figure 2b). In cases where the area of the studied CSRR was smaller than the cross section of the waveguide, an appropriately constructed metallic mask with a rectangular opening matched to the CSRR dimensions was used to cover the empty space between the CSRR and the walls of the waveguide, giving special attention so that the studied sample is placed exactly at the center of the mask and consequently being in the middle of the waveguide cross section. In such a way, any measuring inconsistencies regarding the position of the sample in the waveguide can be minimized. During measurements, the loaded CSRR was carefully placed on the effective area of the waveguide, in a centrally aligned manner in both orientations (TE and TM), ensuring that the maximum intensity of the transmitted signal passes through the examined CSRR. As described above, the precise positioning ensured via the custom-cut metallic slide fills any remaining space unoccupied by the CSRR, thus providing reproducible measurement conditions.

**Figure 2:**
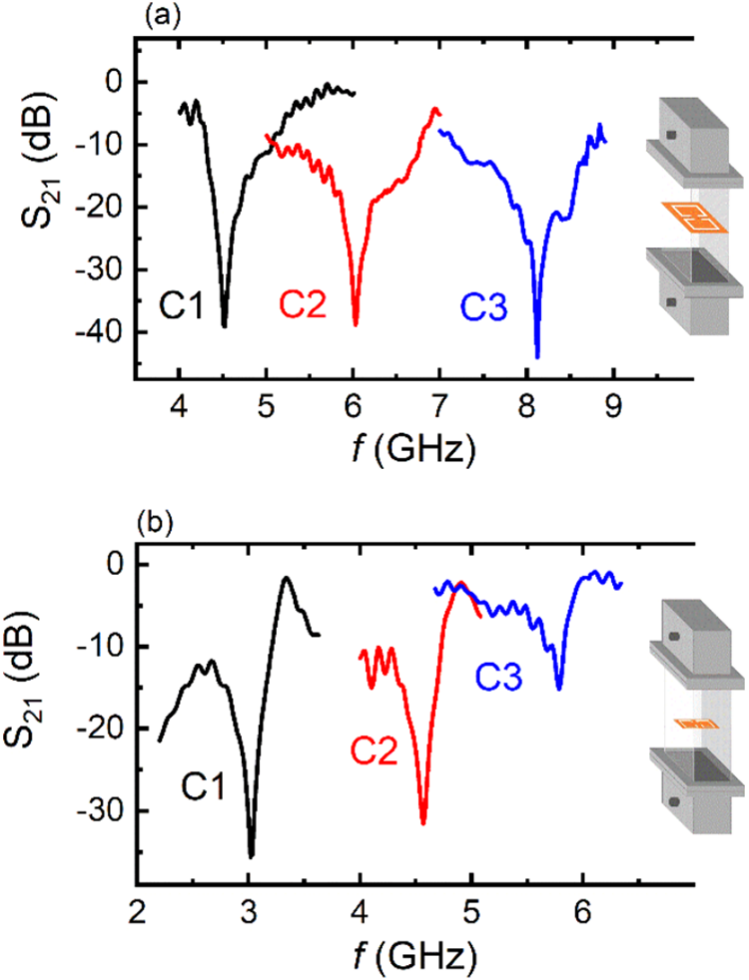
S_21_ vs frequency spectra of all studied CSRRs in (a) TE and (b) TM orientation, respectively. Insets in both panels show the orientation of the CSRR into the waveguide.

### 2.3 Theoretical Simulations

The obtained experimental results were additionally supported by theoretical simulations. Electromagnetic simulations of the proposed designs were conducted via the Frequency Domain Solver of the CST Studio Suite (CST Microwave Studio, Computer Simulation Technology GmbH, Darmstadt, Germany), using continuous wave (CW) excitation and exploiting the finite element analysis (FEA) method. The simulated topology was thoroughly modeled, entailing two CSRRs with opposing gaps, with respect to the material properties of the PCB layout, namely, an FR-4 substrate (ε_r_ = 4.8, tanδ = 0.017) with copper metallization (σ = 5.96 × 10^7^ S/m). Perfect electric conductor (PEC) boundary conditions were applied to all waveguide walls, where the resonator was excited through the TE_10_ mode of the waveguide. The corresponding S-parameters in the selected frequency range were extracted by using rectangular waveguide ports.

### 2.4 Metasurface Sensing Properties

In order to study the sensing properties of the CSRRs, with respect to different types of honey (i.e., chestnut honey (CH), pine honey (PH), and orange blossom honey (OH)), microwave transmission experiments were conducted using the experimental setup, as shown in Figure 1c.^43^ For the purposes of this experiment, only a tiny amount of honey must be inserted into the engraved area of the CSRR. Nevertheless, due to the high viscosity of the honey, filling the engraved CSRR area cannot be uniform, which would be reflected in the recorded transmission spectrum. To overcome this challenge, water solutions of honey were prepared, i.e., 10 mL from each type of honey was diluted with 2.5 mL of deionized water, in glass vials, under magnetic stirring at 310 rpm for 30 min at *Τ* = 50 °C. Traces of those solutions are inserted in the CSRR-engraved area. In general, it should also be stressed that the volume of the sample inserted into the CSRR-engraved path can heavily influence its electromagnetic behavior. In particular, increasing the sample volume tends to lead to shallower resonance dips followed by a concurrent red shift of resonance frequency.^29^ Hence, all of the experiments were performed with half of the engraved area filled with the corresponding liquid. Such an amount of liquid is appropriate to give reasonable EM signal changes, while it is also enough to ensure its homogeneous distribution into the engraved area. To control the liquid volume inserted into the CSRR, a micropipet with a resolution of 0.2 μL was used, ensuring the constraint of the liquid into the engraved path of the CSRR. Then, the loaded CSRR was carefully inserted into the waveguide, which had previously been turned into its vertical position. Thus, the sample under testing can be placed horizontally, preventing the liquid from splitting out of the engraved area. Notably, the presence of water in the solution will definitely affect the electromagnetic response of the CSRR, and it might partially mask its response with respect to the honey. Nevertheless, the presence of water in all three solutions will equally affect the EM behavior of the CSRR, so any difference could safely be attributed to the different honey types. Moreover, it should also be noted that even slight deviations regarding the homogeneity during sample placement would definitely have a significant impact on the electromagnetic properties of the CSRR. In order to address such concerns, preliminary experiments were conducted by introducing a fixed volume of a sugar–honey solution into the CSRR and subsequently recording its electromagnetic response with respect to the previously described setup (Figure 1c). The aforementioned procedure was performed several times, while, during measurement intervals, the CSRR was thoroughly rinsed with distilled water and dried using flowing nitrogen. Corresponding statistical analysis of the collected transmission spectra demonstrated a standard deviation in the range of 2–5%, with respect to the mean values, ensuring the reproducibility of the experimental results.

Furthermore, the sensing properties of the CSRRs were studied in the presence of a sweetener (sugar or maple syrup) in the honey. In general, sweeteners are widely used, replacing corresponding amounts of honey, leading to honey adulteration and a consequent quality reduction.^44^ As previously described, several honey/water solutions were made, enriched with the selected sweetener, as seen in Table 2. Special attention has been paid so that the amount of the added sweetener replaces the same amount of honey. Then, an appropriate amount of those solutions was inserted into the engraved area of the CSRR, and the corresponding transmission spectra were recorded.

**Table 2:** Code Name, Included Substances, and Volume Concentration of the Aqueous Solutions Used as Sensing Materials

**solution name**	**solution ingredients**	**ratio**	**additive concentration** (% w/w)
**CW**	chestnut honey/water	10 gr/2.5 mL	20
**PW**	pine honey/water	10 gr/2.5 mL	20
**OW**	orange honey/water	10 gr/2.5 mL	20
**HWSG**	honey/water/sugar	8 gr-m_sugar_/2 mL/m_sugar_	0–30
**HWSR**	honey/water/syrup	8 gr/2 mL/2 gr	30
**HWP**	honey/water/PET	16 gr/4 mL/2 gr	10
		8 gr/2 mL/2 gr	20

Finally, the EM behavior of the CSRR is studied against honey solutions containing poly­(ethylene terephthalate) (PET) microparticles. Such polymers are commonly used in food packages, especially in honey;^18^ therefore, it is prudent to explore any probable contamination coming from plastic packaging.^19^ In this context, honey/water solutions were prepared, as previously, in which PET microparticles were added at two different nominal concentrations, i.e., 10 and 20% (w/w). Each solution was sieved through metallic filters of different mesh porosities, namely, 40, 50, 60, and finally 100 meshes, so that the mean size of PET particles in the final filtrate is ∼149 μm. The filtrated solutions were EM examined using the CSRRs. Corresponding solution code names, concentrations, and other information are included in Table 2.

### 2.5 Spectroscopic Characterization

All of the above-described honey solutions were characterized by employing Raman, FTIR, and dielectric spectroscopy experiments. Raman measurements were conducted at room temperature using a confocal Raman microscope (LabRAM HR; HORIBA FRANCE SAS, Lille, France). Raman excitation was achieved with a laser line centered at 785 nm due to the fluorescence of the honey samples. A 600 grooves/mm grating resulted in a Raman spectral resolution of around 2 cm^–1^. Each honey sample drop was observed and measured under the Raman microscope with a 10× (MPlan 10×/0.25, OLYMPUS Corporation, Tokyo, Japan) objective lens. At the edge of each honey drop, the *z*-axis was zeroed out, and the measurements were conducted at 1400 μm in the center of each honey drop. For each sample, three measurement points were taken with an acquisition time of 20 s, 5 accumulations per point, 115 mW laser intensity (maximum power 100% on the samples), and a spectral range of 200–1850 cm^–1^. Processing and analysis of the acquired raw Raman spectra were achieved through the instrument’s original software (LabSpec, version LS6; Horiba, Lille, France). Initially, a smoothing under a Gaussian filter with a kernel of five points (denoise at 5) was used, where cosmic rays were removed, while the background was removed using a baseline correction at the sixth-order polynomial function. Finally, a shift to zero was applied with a unit vector. All of the averages were generated through OriginLab software (OriginPro 2021; Originlab Corporation, Northampton, MA). For PET MPs identification, the Peak Finder was used.

In addition, ATR/FT–IR (absorbance) experiments were carried out using a Bruker Vertex 70v FT–IR vacuum spectrometer, equipped with an A225/Q Platinum ATR unit with a single reflection diamond crystal, which allows the infrared analysis of unevenly shaped solid samples through total reflection measurements, in a spectral range of 7500–350 cm^–1^. A broadband KBr beamsplitter (Bruker Optik GmbH, Rosenheim, Germany) and a room-temperature broadband triglycine sulfate (DTGS) detector (Bruker Optik GmbH, Rosenheim, Germany) were used, while interferograms were collected at 4 cm^–1^ resolution (8 scans), apodized with a Blackman–Harris function and Fourier transformed with two levels of zero filling to yield spectra encoded at 2 cm^–1^ intervals. Before the samples were scanned, a background diamond crystal was recorded, and each sample spectrum was obtained by automatic subtraction of it. For each measurement, 2 μL of each honey sample was carefully placed on the ATR sample compartment without the use of the ATR press, while after every measurement, the sample area of the A225/Q ATR unit was cleaned with pure ethanol (Et–OH; Sigma-Aldrich, Munich, Germany).

Finally, the dielectric permittivity of all honey solutions was measured by employing the standard coaxial probe method. An open-ended coaxial probe (N1501A dielectric probe kit, Keysight Technologies, CA) was connected to the VNA (P9372A Streamline Vector Network Analyzer, Keysight, California). The probe was immersed directly in each honey solution, and the dielectric permittivity values (real and imaginary parts) were automatically calculated through the VNA software (N1500A Materials Measurement Software Suite, Keysight, California) in the frequency regime 1–9 GHz.

## 3 Results and Discussion

### 3.1 Electromagnetic Properties of the Metasurfaces

Figure 2 shows the EM response for all studied CSRRs, for both TE (Figure 2a) and TM orientations (Figure 2b).

Strong, well-defined drops of the S_21_ magnitude are observed for all CSRRs, at certain frequencies, in both orientations, indicating a well-established resonance behavior. Considering the absence of any feature in the corresponding reflection spectra (not shown here), the observed minima can be attributed to the absorption of the incident wave. Therefore, all CSRRs resonate at certain frequencies, in agreement with the corresponding theoretical simulations as depicted in Figure S1. Regardless of the measurement orientation, it is seen that the resonance frequency increases with the CSRR’s size decrement. Such a behavior is identically consistent with previous studies.^29,40^ Hence, the CSRRs studied here exhibit a significant EM response.

### 3.2 Sensing Performance of the Metasurfaces: Honey Type Discrimination

In order to clarify any distinguishable EM response of the engraved CSRRs, in the presence of a honey solution, appropriate transmission experiments were performed. In particular, an appropriate amount of CW solution was inserted into the CSRR-engraved area, and the corresponding S_21_ vs *f* spectrum was recorded. Experimental results for all CSRRs are listed in Figure 3. It is clearly seen that, in all cases, the presence of the CW solution results in a sizable shift of the resonance, toward lower frequencies, while the S_21_ magnitude also decreases. In the TE orientation, the shift is ∼285 MHz for the S1 sample, while it decreases to ∼40 MHz for the S3 sample (i.e., Figure 3a,c,e). Thus, one can easily see that the resonance shift decreases with decreasing CSRR size. Such a behavior is opposite to the trend observed for engraved CSRRs, proposed for water quality control.^29^ Even more, in the TM orientation, the corresponding shift seems to be unaffected by the size of the CSRR; however, all curves become shallower, possibly due to the lower contribution of the electric field component in the TM orientation combined with the existence of higher dielectric losses of the sample (Figure 3b,d,f).

**Figure 3:**
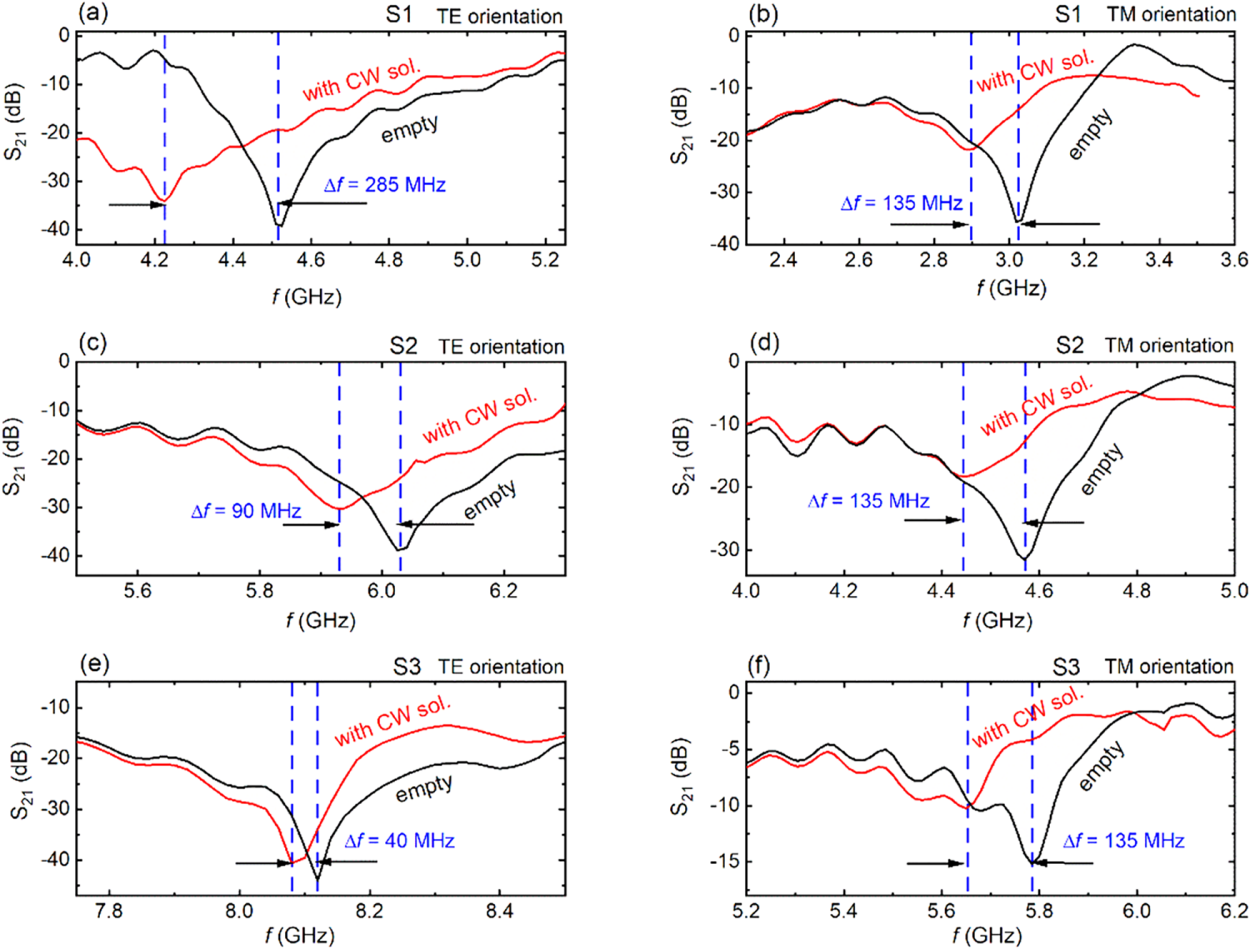
S_21_ vs *f* curves with (red line) and without (black line) honey solution into the engraved area of the (a) S1 CSRR in TE orientation and (b) TM orientation. Corresponding S_21_ vs *f* graphs with (red line) and without (black line) honey solution into the engraved area of the (c) S2 CSRR in TE orientation and (d) TM orientation. Finally, S_21_ vs *f* spectra with (red line) and without (black line) honey solution into the engraved area of the S3 CSRR in (e) TE orientation and (f) TM orientation.

Despite the differences, experimental evidence shows the EM response of all CSRRs, which is tuned by the presence of a honey solution. Thus, it becomes of great interest to explore their EM response in the presence of solutions containing various honey types. In this context, transmission spectra, upon the presence of SW, PW, and OW solutions, are demonstrated in Figure 4 for the S2 CSRR. Particularly, discrete transmission spectra are observed for different honey solutions in both measured directions (Figure 4a,c). Considering that all solutions include the same concentration of water (i.e., Table 2), it can be safely assumed that the observed differences are attributed to the different types of honey.

**Figure 4:**
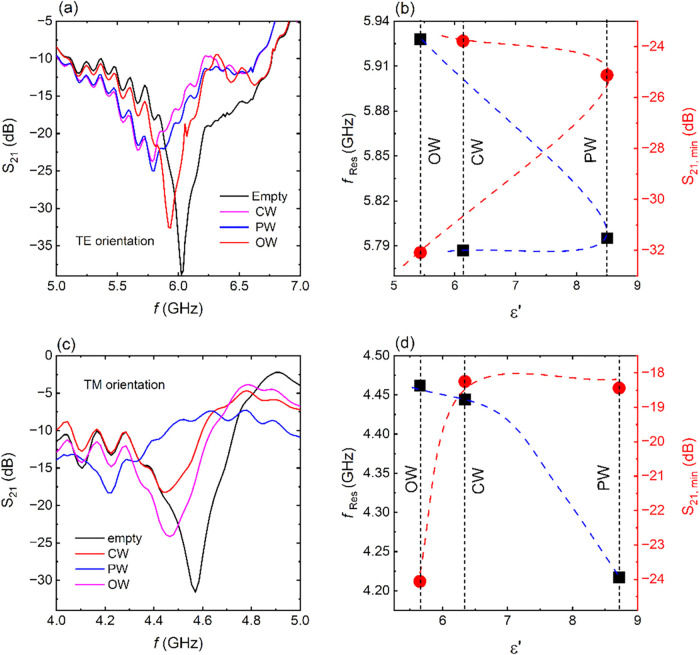
S_21_ vs frequency for various kinds of honey for S2 CSRR in the (a) TE orientation and (b) resonance frequency *f*_Res_ (black symbols) and corresponding S_21_ value (red symbols) with respect to the dielectric permittivity. Blue and red dashed lines are guides to the eye. They also show the evolution of both the evolution of the *f*_Res_ and the S_21_ magnitude, as extracted from panel a. Black dashed lines show the dielectric permittivity of each honey solution. (c) S_21_ vs frequency for various honey types, in the TM orientation. (d) Resonance frequency *f*_Res_ (black symbols) and corresponding S_21_ value (red symbols) with respect to the dielectric permittivity. Blue and red dashed lines are guides to the eye. They also show the evolution of both the evolution of the *f*_Res_ and the S_21_ magnitude, as extracted from panel a. Black dashed lines show the dielectric permittivity of each honey solution.

A detailed look at Figure 4a shows that the CW curve resonates at a lower frequency than the PW curve although the PW sample has a greater dielectric permittivity value than the CW, as shown in Figure 4b. Moreover, the S_21_ intensity between CW and PW curves slightly differs, which cannot be correlated to the large difference in their permittivity values. Even more, the evolution of both the resonance frequency and the S_21_ intensity, with respect to the dielectric permittivity (Figure 4b), is nonmonotonic. Thus, experimental results come in contrast with the RLC model, previously discussed, indicating a possible inability of the CSRR to distinguish among different types of honey in the TE orientation. On the other hand, resonance frequencies in the TM orientation monotonically decrease with increasing ε′ (Figure 4d), indicating the distinction among the different honey types. Therefore, the hereby studied CSRRs not only react in the presence of honey solution but are capable of qualitatively distinguishing among different types of honey, when a proper orientation of measurement is chosen.

The sensing performance of the studied CSRRs is determined through their quality factor *Q*, their relative sensitivity *S*, and their Figure of Merit *FoM*, through the following relations:

1 $$Q=\frac{f_{res}}{\mathrm{fwhm}}$$

2 $$S\left(\%\right)=100\times \frac{f_{\mathrm{empty}}-f_{\mathrm{honey}}}{f_{\mathrm{empty}}\times \left(\varepsilon_{\mathrm{honey}}^{\prime }-1\right)}$$

3 FoM=S/fwhm

where *f*_res_ is the resonance frequency, fwhm is the full width at half-maximum of each electromagnetic minimum, *f*_empty_ is the resonance frequency of empty CSRR, *f*_honey_ is the resonance frequency of the CSRR with honey solution included, and ε′_honey_ is the dielectric permittivity of the honey solution. Calculated *Q*, *S*, and *FoM* values are presented in Table 3 for all studied CSRRs in all measured orientations. All quantities have been calculated with respect to the CW solution, while similar results are obtained for both PW and OW solutions as well (Supporting Information Tables S1 and S2). It is seen that S3 CSRR shows the highest *Q* values in both measurement orientations. In particular, the highest *FoM* value is obtained for the S3 sample in the TM direction. In general, the *Q*, *S*, and *FOM* values for all samples in the TE direction are affected by the sample dimensions. The decreased CSRR performance in the TE direction mainly comes from the reduced resonance frequency shift, upon decreasing CSRR size (i.e., see Figure 3a,c,e), which affects the corresponding sensitivity magnitude. In addition, all S_21_ curves are broad; thus, fwhm is enhanced, resulting in reducing the *FoM* values. On the contrary, in the TM orientation, frequency shift is almost unaffected by the CSRR size. Thus, the sensitivity values are kept constant, leading to almost unchanged *FoM* values (although broad, the S_21_ curves exhibit similar fwhm levels). Therefore, the TM direction arises as the most effective, as far as the CSRRs’ sensing capability with respect to the honey type. Hence, transmission measurements will be conducted in TM orientation for the rest of this study.

**Table 3:** Quality Factor *Q*, Sensitivity *S*, and Figure of Merit *FOM* Values for all CSRRs Containing CW Solution in Both Measurement Orientations

**orientation**	**CSRR**	***f***_**empty**_ **(GHz)**	***f***_**honey**_ **(GHz)**	**ε′**_**honey**_	**fwhm**	***Q***	***S*** **(%)**	***FoM***
**TE**	**S1**	4.510	4.225	10.69	0.435	9.70	0.65	1.50
	**S2**	6.025	5.935	9.81	0.270	21.98	0.17	0.63
	**S3**	8.120	8.080	9.43	0.190	42.53	0.06	0.31
**TM**	**S1**	3.019	2.884	10.98	0.261	11.05	0.45	1.72
	**S2**	4.570	4.435	10.62	0.210	21.12	0.31	1.46
	**S3**	5.785	5.560	10.40	0.215	25.86	0.41	1.92

Comparison among the hereby studied CSRRs and other resonator-based sensors reported in the literature is shown in Table 4. It is seen that the proposed CSRR structures exhibit low *Q* values, most likely due to the broad profile of the S_21_ curves (Figure 3). However, their sensitivity is comparable or even superior to the corresponding sensitivities of other sensors listed. Accordingly, the obtained results are comparable to other microwave honey quality sensors.^45,46^ In other words, the resonance dips are distinct; nevertheless, they are quite broad, resulting in a reduced performance of the proposed CSRR sensors. Thus, optimization of these should be considered. As shown in Figure 1a, the studied CSRRs show some weak construction issues such as rather round corners, rounded-edged gaps, and deviations of the rectangular topology upon decreasing CSRR size. Improvement of those construction issues would result in the sharpening of the S_21_ dips and consequently in the enhancement of their quality factor, improving their overall sensing performance. Even though experimental results clearly demonstrate the capability of the studied CSRRs to distinguish among different types of honey, suggesting their strong candidacy for honey-sensing applications.

**Table 4:** Comparison among the Hereby Studied CSRRS and Other Resonators Proposed for the Characterization of Liquid and Semi-Liquid Materials

**refs**	***f***_**res**_ **(GHz)**	**topology**	***Q***	***S*** **(%)**	***FoM***	**SUT**
this study (sample S1 TM orientation)	3.019	CSRR	10.9	0.45	1.72	honey
^24^	5.3	SRR		4.64		liquids
^25^	6	SRR	38	0.17		milk
^27^	2.45	MCSRR	31			liquids
^28^		CSRR		0.50		water
^29^	3.6	CSRR	90.7	0.034	0.001	water
^41^	8.2	CSRR	97.8	1.19	0.14	oil
^47^	2.5	CSRR	520	0.29		liquids
^48^	2.5	CSRR	520	0.20		semisolids
^49^	2.5	CSIW	700	0.12		semisolids

### 3.3 Sugar Adulteration

Next, we proceeded with further experimental studies focusing on the adulteration of honey. As a first step, we studied the effect of sugar inclusion in pure honey. Thus, in solutions of pure honey, traces of sugar are added, so adulterated honey solutions are obtained. Detailed information about the prepared honey/sugar solutions is listed in Table 5.

**Table 5:** Solution Name, Sugar Concentration, and Honey/Water Volumes of the Solutions

**solution**	**sugar content** (% w/w)	**honey content** (gr)	**water content** (ml)
**HWSG0**	0	8.0	2
**HWSG1**	5	7.5	2
**HWSG2**	10	7.0	2
**HWSG3**	15	6.5	2
**HWSG4**	20	6.0	2

Traces of those solutions are included in the S1 CSRR, and the corresponding EM behavior is recorded, in TM orientation. Experimental results are shown in Figure 5. Here, it has to be stressed that changing CSRRs from S2 to S1 does not have any impact on the measurement since all three samples exhibit comparable performance (i.e., Table 3).

**Figure 5:**
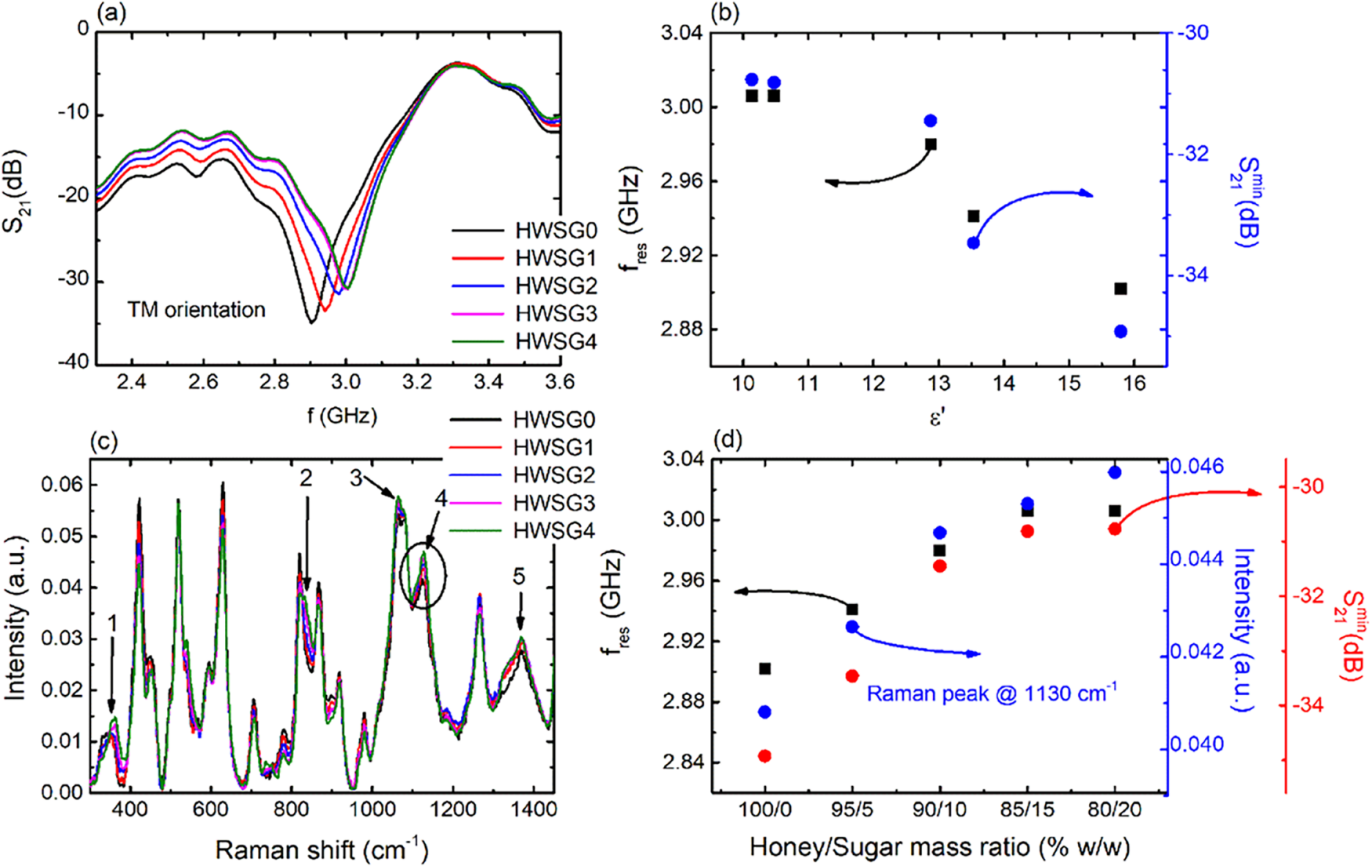
(a) S_21_ vs frequency for honey solutions containing various sugar concentrations, as measured for S1 CSRR in TM orientation. (b) Resonance frequency and dip intensity as a function of the measured dielectric permittivity for all sugar-adulterated samples. (c) Raman spectra of all honey samples adulterated with sugar. Characteristic peaks indicate a distinction between samples of different sugar content. (d) Progression of resonance frequency (black symbols), S_21_ magnitude (red symbols), and 1130 cm^–1^ Raman peak (blue symbols), for different sugar contents.

As shown in Figure 5a, the resonance frequency shifts to higher values with increasing sugar concentration, while a reduction in the S_21_ intensity is observed. Therefore, a monotonic EM response is observed in both the resonance frequency change and the S_21_ intensity, upon the increment of the sugar inclusion, indicating the capability of the CSRR to detect sugar adulteration in honey. Notably, the presence of sugar can be detectable for concentrations as low as 5% w/w. The EM response of the S1 sample can be correlated to the dielectric permittivity of honey solutions. Dielectric permittivity monotonically decreases with increasing sugar concentration due to the low permittivity values of sugar in the microwave region^50^ (i.e., Supporting Information Figure S6). Interestingly, both *f*_res_ and S_21_ magnitude follow a similar monotonic trend, as pictured in Figure 5b, indicating the capability of the CSRRs, to quantitatively detect the sugar adulteration in honey. Even more, the EM behavior of the CSRRs is directly compared to the corresponding Raman spectra of the adulterated solutions, as shown in Figure 5c. The addition of sugar can be identified from the denoted characteristic peaks 1, 2, 3, 4, and 5, which are correlated to the vibrations of sucrose and fructose, the main contents of sugar (full peak assignment is presented in Supporting Information Table S3). The intensity of those peaks gradually increases with respect to the sugar loading. In particular, the characteristic peak, located at 1130 cm^–1^, is closely linked to the presence of sucrose, denoting the δ­(COH) vibrational state in sucrose aqueous solution.^7,51^ Hence, the corresponding peak intensity could be exploited as an indicator for quantifying the included sugar in the adulterated solution. The evolution of the 1130 cm^–1^ peak intensity can be shown in Figure 5d (blue circles), which increases with increasing sugar loading. In comparison to that, the corresponding resonance frequency increases, while the S_21_ magnitude decreases, following the trend of the Raman peak. Hence, both resonance frequency and S_21_ magnitude trends closely resemble the behavior of the corresponding Raman spectra, suggesting that the CSRR essentially detects and quantifies honey adulteration quite effectively. Notably, similar observations are obtained when comparing EM with corresponding FTIR spectra (i.e., Supporting Information Figure S9 and Table S4). Therefore, the EM behavior of the CSRR is fully consistent with state-of-the-art spectroscopic evidence, coming from FTIR and Raman experiments, highlighting its sensing performance in the presence of sugar adulteration in honey.

Here, it has to be noted that the study focuses on the assessment of the sensing performance of the CSRRs as a honey quality evaluation tool. In this context, Raman spectroscopy was employed as a complementary technique for cross-validating the electromagnetic response of the CSRRs. On the other hand, an in-depth analysis regarding the interdependence of the molecular structure and dielectric features could be interesting although it sounds like it is out of the scope of this study.

### 3.4 Discrimination among Different Adulterants

Then, we tested the sensing properties of the CSRRs among different adulterating substances. For this reason, we opted for maple syrup, which is another common artificial sweetener. In order to study its effect on the EM behavior of the CSRRs, two different adulterated honey solutions were made: one with maple syrup (which is marked as HWSR) and another with sugar (marked as HWSG). In both solutions, the sweetener is introduced at 20% w/w into a pure honey solution. An appropriate amount of these solutions is placed into the engraved area of the S2 CSRR, and its EM response is measured in the TM orientation. Corresponding experimental results are provided in Figure 6.

**Figure 6:**
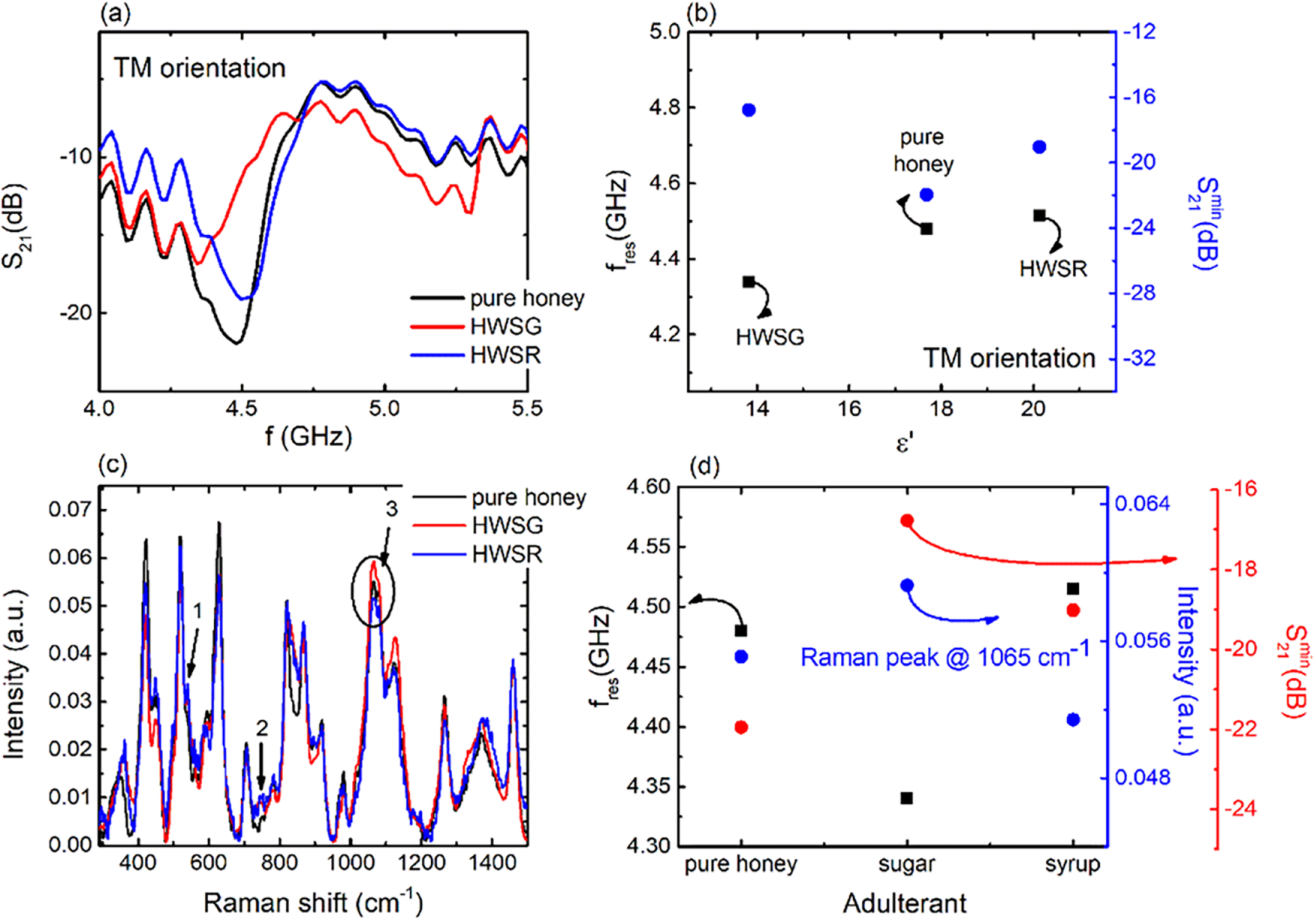
(a) S_21_ vs frequency for honey solutions containing different adulterating substances at a fixed concentration of 30% (w/w), as measured for the S2 CSRR in TM orientation. (b) The dependence of resonance frequency and intensity dip on the dielectric permittivity for all honey solutions containing different adulterants. (c) Raman spectra of all honey samples impurified with different adulterating substances. Characteristic peaks denoting a distinction between different adulterants are numbered. (d) The evolution of resonance frequency (black symbols), S_21_ magnitude (red symbols), and Raman peak at 1065 cm^–1^ (blue symbols) for all adulterated honey samples.

As can be seen from Figure 6a, distinct S_21_ spectra are obtained among pure honey solutions and those containing sweeteners. All spectra show resonance behavior; however, the HWSG curve is red-shifted while the HWSR is blue-shifted, with respect to the S_21_ curve of the unadulterated solution. Moreover, both HWSG and HWSR solutions show decreased S_21_ magnitude compared to the pure honey solution. The above observations are also corroborated by corresponding dielectric permittivity measurements (i.e., Supporting Information Figure S7). First, the HWSG solution exhibits a lower dielectric permittivity value than pure honey, which coincides with the red shift of the resonance frequency. In contrast, the HWSR solution resonates at a higher frequency than the pure one, which is attributed to the fact that the HWSR solution shows the highest dielectric permittivity value among all adulterants. Therefore, the resonance frequency varies monotonically with dielectric permittivity (Figure 6b) and thus with the adulterant type, giving more credence to the sensing capabilities of the engraved CSRRs.

Moreover, the above results are compared to the corresponding Raman spectra, as shown in Figure 6c. All Raman spectra exhibit a similar pattern due to the same type of honey used as a primary material and exist in the highest concentration; nonetheless, the presence of adulterating substances is still distinguishable. In particular, honey adulteration can be discriminated by the indicated peaks 1, 2, and 3 of Figure 6c. Maple syrup adulteration can be identified from Raman peaks 1 and 2 in the HWSR solution, while the presence of sucrose can be identified by peak 3 in the HWSG solution; the scenario of sugar adulteration has been extensively discussed in the previous section. Fully assigned peaks are listed in Supporting Information Table S5. Moreover, as the Raman peak at 1065 cm^–1^ corresponds to the presence of sucrose in adulterated honey solutions,^52^ its intensity variations could be exploited as an indicator for the detection of sugar-based additives. Based on that, Figure 6d describes the evolution of resonance frequency vs 1065 cm^–1^ peak intensity, with respect to the included adulterant. In general, changes in the peak intensity seem to be correlated with the observed variations in resonance frequency and dip intensity of the samples, cross-validating the sensing performance of the CSRR. Notably, a similar trend is shown when comparing EM and FTIR spectra (i.e., Supporting Information Figures S10a,b and Table S6). Based on the above, the validity of the proposed method regarding the detection of commonly used sweeteners for the adulteration of honey could be safely presumed.

### 3.5 Microplastic Contamination

Another parameter examined regarding the sensing capability of the CSRRs concerns the microplastic contamination of honey. Particle migration from plastic packaging to food constitutes a rapidly increasing concern. Although little is known about the total impact of plastic pollution on human health, microplastics can accumulate in human organs and penetrate cell membranes, leading to serious health issues.^53^ Lately, the migration of microplastics in honey through food packaging has been realized through comprehensive spectroscopic studies.^19^ Considering that microplastics demonstrate distinct dielectric characteristics, there is potential to identify such contaminants through the transmission of low-power microwaves. Hence, the possibility of detecting contaminated honey samples with the use of CSRRs would be of great significance. For this reason, several honey solutions were prepared, including specific quantities of PET microparticles (MPs). Detailed information about the prepared solutions is listed in Table 6.

**Table 6:** Solution Name and Nominal Concentration of all PET Contaminated Honey Solutions Used in This Study

**solution**	**PET content** (% w/w)	**honey content** (gr)	**water content** (ml)	**ε′**_**solution**_
**HWP0**	0	8.0	2	21.92
**HWP1**	10	16.0	4	23.18
**HWP2**	20	8.0	2	22.62

A fixed amount of those solutions is placed into the engraved area of the S2 CSRR, and its EM response is recorded in TM mode. Corresponding experimental results are shown below (Figure 7).

**Figure 7:**
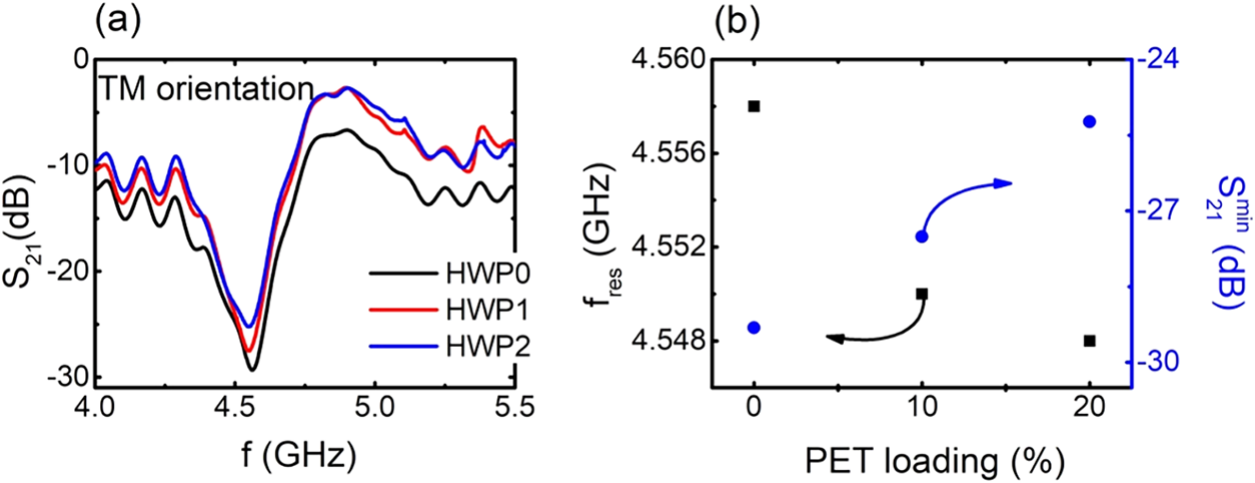
(a) S_21_ vs frequency for honey solutions contaminated with PET MPs at different concentrations. (b) The dependence of resonance frequency and transmission peak intensity on PET concentration.

As depicted in Figure 7a, the EM response of the S2 CSRR exhibits a clear distinction regarding the presence of MPs in honey. The resonance frequency of the solution shifts toward lower values in the presence of PET, while at the same time, the S_21_ intensity is clearly reduced. This is indeed represented in Figure 7b, where HWP0 exhibits resonance with the highest intensity dip. The addition of MPs had a detectable impact on the resonance frequency and the S_21_ magnitude. Interestingly, microplastic contamination cannot be detected for loadings lower than 10% due to constraints related to the dimensions of the PET particle size and the length of the incident wave.^54^ In this case, the performance of the CSRRs seems to be poor. Although the quantification of plastic contaminants in the microscale was not achieved, the EM response of the CSRR could be a good marker denoting the presence of microplastics in honey. On top of that, given the fact that there is very limited knowledge on how microwave sensing can be applicable for the detection of MPs in honey, the denoted sensing ability of the CSRR can be proven essential for future research activity.

## 4 Summary and Conclusions

In this study, millimeter-scale engraved rectangular CSRRs were tested as potential microwave sensors for honey quality control. The EM response of the CSRRs was investigated through transmission experiments in the microwave regime in both TE and TM modes. All CSRRs demonstrate well-defined transmission minima at specific frequencies, being in agreement with the corresponding analytical models.

The EM response of the CSRRs was examined in the presence of honey solutions of different origins. The introduction of the aforesaid solutions into the engraved area of CSRRs causes a conspicuous shift in resonance frequency toward lower values, depending on the honey type. All absorption peaks become shallower in the presence of honey. For all loaded CSRRs, a sharper resonance is obtained in the TM orientation. The sensitivity and *FοM* values are found to be inferior compared to other metamaterial-based honey sensors. However, the behavior of the transmission spectra is consistent with the corresponding spectroscopic results, indicating the ability of the CSRRs to discriminate different types of honey.

Furthermore, the sensing performance of the CSRRs in detecting various amounts of sugar introduced into commercial honey as an adulterant was analyzed. Based on the experimental transmission spectra, as recorded in TM orientation, resonance frequencies and equivalent frequency shifts were extracted and used thereafter to differentiate solutions of discrete sugar concentrations. Resonance frequency increases with respect to sugar content, revealing a proportional dependence on dielectric permittivity. A decreasing trend in the intensity of the absorbance peaks is observed as sugar concentration increases, providing the feature of quantitative sensing to the CSRR. Such a behavior is in total agreement with the complementary spectroscopic results. A slightly increased *FOM* is obtained for this case although still low. Nonetheless, reported measurements have shown sufficient sensitivity, adequate for practical adulteration monitoring in honey quality control.

In addition, the discriminatory ability of the CSRRs among different adulterating substances, sugar and maple syrup, was tested. Several honey solutions containing commonly encountered adulterants were prepared, and the EM response of the mid-dimensional CSRR was examined in TM orientation. Corresponding transmission spectra reveal a distinct shift in the resonance frequency according to the included substance. Absorption peaks demonstrate decreased intensity for all adulterated solutions, while corresponding spectroscopic and dielectric permittivity spectra also exhibit noticeable differences in the obtained characteristic peaks.

Finally, the sensing performance of the CSRRs regarding microplastic detection was also investigated. By the introduction of honey solutions contaminated with PET MPs into the engraved area of the CSRR, a sizable change in its resonant response occurs. Although the quantification of microplastics was not achieved, the discrete EM response of the CSRR could effectively be exploited as a contamination marker. The above experimental findings indicate that mechanically engraved metasurfaces can potentially be applied as sensors regarding honey quality control. In addition, even if the present study focuses on the investigation of liquid samples, the dielectric sensing mechanism of the CSRRs can also be extended to samples of a different phase, such as semisolid materials, making their study via the incorporation of CSRRs also feasible. Semisolid materials constitute a category of rising interest in numerous applications, such as food quality monitoring, industrial processes, or even biological analysis, as corroborated by recent studies.^47–49^ Therefore, the integration of metamaterials in dielectric sensing technologies could significantly increase the designing flexibility of sensors and dramatically enhance their overall performance, offering an undoubtedly robust tool in the broad industry of food quality control.

## Supplementary Material



## Notes

**Figure S1.** Corresponding theoretically simulated S_21_ over frequency spectra for all studied CSRRs in (a) TE and (b) TM orientation. **Figure S2**. (a) Electric and (b) Magnetic Field distribution for the studied CSRR topology in TE orientation. **Figure S3**. (a) Electric and (b) Magnetic field energy density of the CSRRs in TE orientation. **Figure S4**. (a) Electric and (b) Magnetic field energy density of the CSRRs in TM orientation. **Figure S5**. (a) Real and (b) imaginary parts of the measured dielectric permittivity as a function of frequency, for various honey samples of different origins. **Figure S6**. (a) Real and (b) imaginary parts of the measured dielectric permittivity for adulterated honey samples at various sugar concentrations. **Figure S7**. (a) Real and (b) imaginary parts of the measured dielectric permittivity for adulterated honey samples containing different adulterants at a fixed concentration. **Figure S8**. (a) Real and (b) imaginary parts of the measured dielectric permittivity for contaminated honey samples containing PET MPs. **Figure S9**. (a) FTIR spectra for all honey samples adulterated with sugar at different percentages. Characteristic absorbance peaks indicating the sucrose presence due to adulteration. (b) Evolution of the absorbance peak No. 2 at 991 cm^–1^, with respect to honey/sugar mass ratio. **Figure S10**. (a) FTIR spectra for honey solutions containing different adulterating substances at a fixed concentration of 30% (w/w). (b) The evolution of resonance frequency and absorbance peak No. 3 at 1030 cm^–1^, with respect to the adulterants used in this study. **Table S1**. Quality factor Q, sensitivity S, and figure of merit FOM values, for PW honey solution, in both measurement orientations. **Table S2**. Quality factor Q, sensitivity S, and figure of merit FOM values, for OW honey solution, in both measurement orientations. **Table S3**. Raman peaks and corresponding assignments of adulterated honey solutions, at various sugar concentrations. **Table S4**. Absorbance peaks and corresponding assignments of adulterated honey solutions, at various sugar concentrations. **Table S5**. Raman peaks and corresponding assignments of adulterated honey solutions containing different adulterating substances at fixed concentrations. **Table S6**. Absorbance peaks and corresponding assignments of adulterated honey solutions containing different adulterating substances (PDF)
