# Supplementary material for: Engraved Microwave Metasurfaces for Potential Application in Honey Quality Control
Source: ACS Omega. 2025 Oct 1;10(40):46780–93. doi: 10.1021/acsomega.5c04380 (PMC12529200; doi:10.1021/acsomega.5c04380)
Supplement: Supplementary file 1 [file ao5c04380_si_001.pdf]

Supporting Information

Engraved microwave metasurfaces for potential application in honey quality control

Argyri Drymiskianaki[1,2,*], Klytaimnistra Katsara[3,2], Vassilis M. Papadakis[4,2], Zacharias Viskadourakis[2] and George Kenanakis[2]

[1.] Department of Materials Science and Engineering, University of Crete, GR-70013 Heraklion, Crete, Greece; adrym@materials.uoc.gr (A.D.)

[2.] Institute of Electronic Structure and Laser (IESL), Foundation for Research and Technology – Hellas (FORTH), N. Plastira 100, Vassilika Vouton, Heraklion GR-70013, Greece; zach@iesl.forth.gr (Z.V.); gkenanak@iesl.forth.gr (G.K.)

[3.] Department of Agriculture, Hellenic Mediterranean University, Estavromenos, GR-71410 Heraklion, Crete, Greece; klyto.katsara@iesl.forth.gr (K.K.)

[4.] Department of Industrial Design and Production Engineering, University of West Attica, GR-12243, Athens, Greece; v.papadakis@uniwa.gr (V.M.P.)

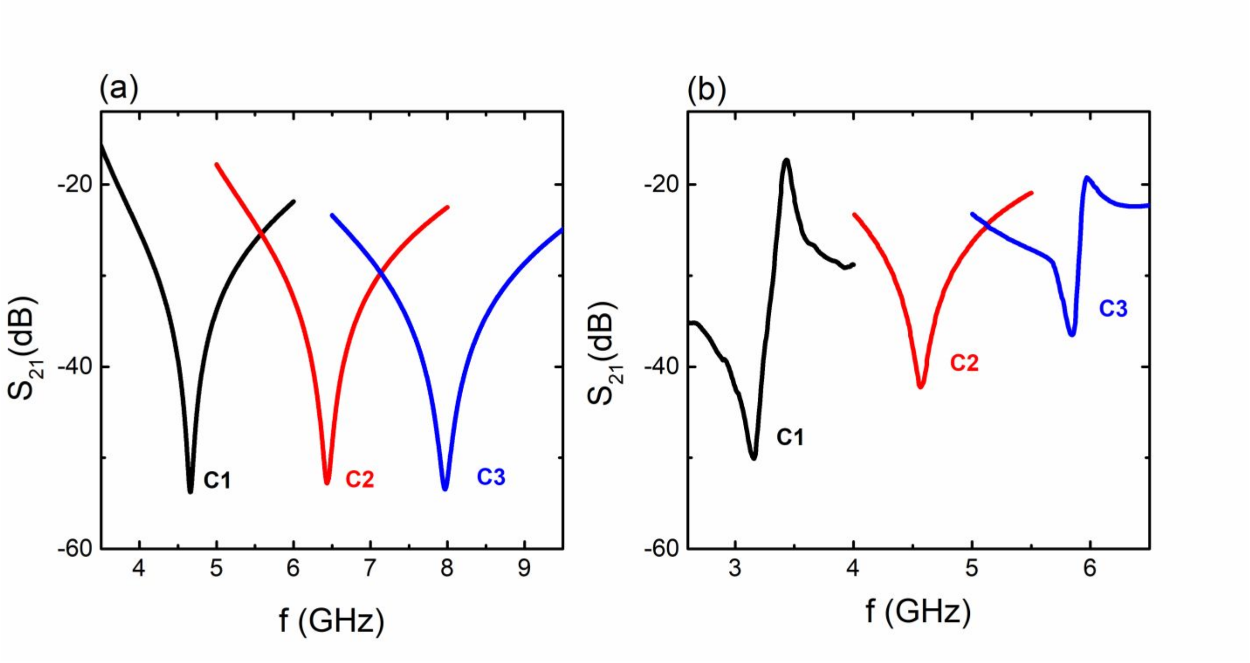

Figure S1. Corresponding theoretically simulated S_{21} over frequency spectra for all studied CSRRs in (a) TE and (b) TM orientation.

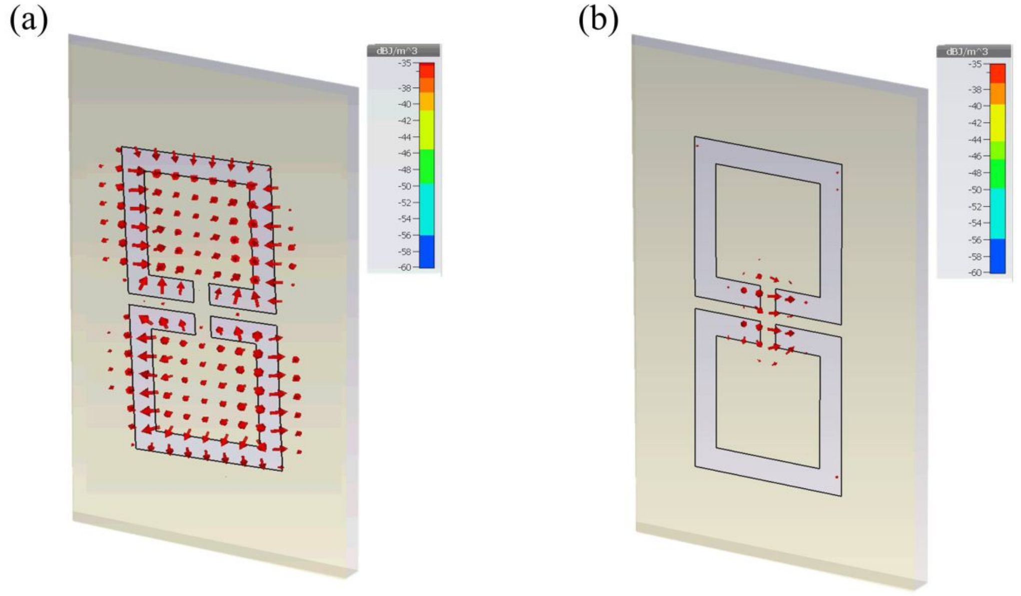

Figure S2. (a) Electric and (b) Magnetic Field distribution for the studied CSRR topology in TE orientation.

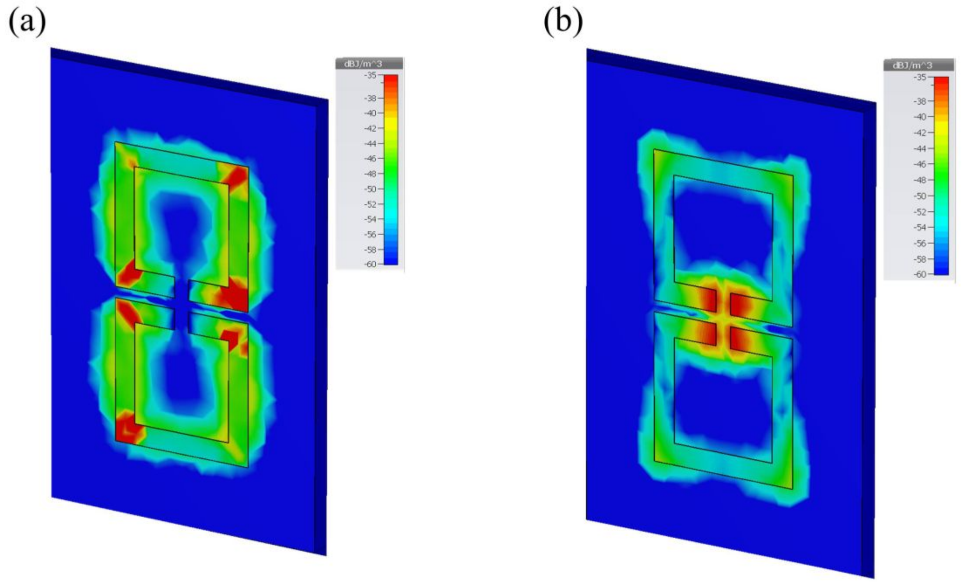

Figure S3. (a) Electric and (b) Magnetic field energy density of the CSRRs in TE orientation.

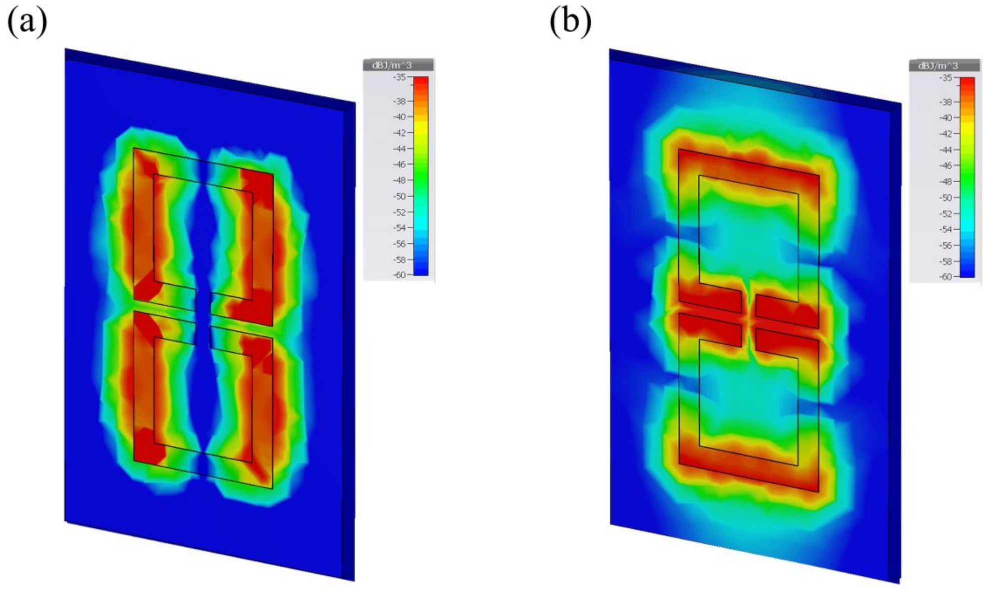

Figure S4. (a) Electric and (b) Magnetic field energy density of the CSRRs in TM orientation.

Table S1: Quality factor Q, Sensitivity S, and Figure of Merit *FOM* values, for PW honey solution, in both measurement orientations.

Orientation	MS	f_{res} (GHz) empty	f_{res} (GHz) honey	ε'_{honey}	FWHM	Q	S (%)	FoM
TE	S1	4.510	4.230	11.64	0.165	25.64	0.58	3.54
	S2	6.025	5.800	10.891	0.345	16.81	0.38	1.11
	S3	8.120	7.840	9.95	0.400	19.60	0.39	0.96
TM	S1	3.019	2.991	11.89	0.171	17.02	0.33	1.92
	S2	4.570	4.210	11.64	0.330	12.76	0.74	2.24
	S3	5.785	5.545	11.43	0.285	19.46	0.40	1.40

Table S2: Quality factor Q, Sensitivity S, and Figure of Merit *FOM* values, for OW honey solution, in both measurement orientations.

Orientation	MS	f_{res} (GHz) empty	f_{res} (GHz) honey	ε'_{honey}	FWHM	Q	S (%)	FoM
TE	S1	4.510	4.075	10.45	0.270	15.09	1.02	3.78
	S2	6.025	5.920	9.58	0.240	24.67	0.20	0.85
	S3	8.120	7.980	8.91	0.360	22.12	0.22	0.61
TM	S2	4.570	4.450	10.52	0.255	17.45	0.28	1.08
	S3	5.785	5.545	10.19	0.450	12.32	0.45	1.00

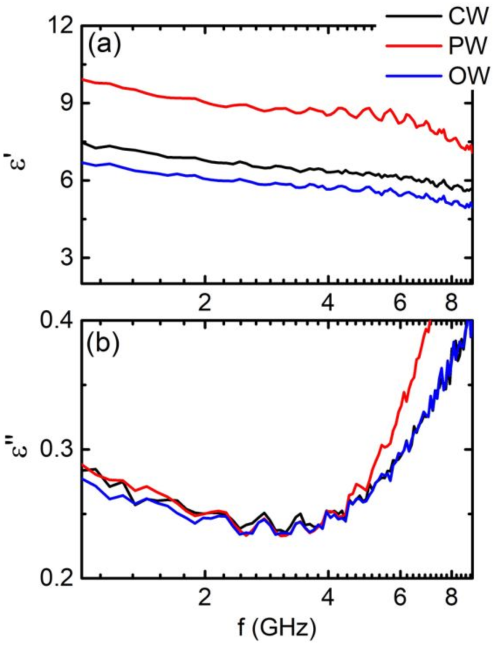

Figure S5. (a) Real and (b) Imaginary part of the measured dielectric permittivity as a function of frequency, for various honey samples of different origins.

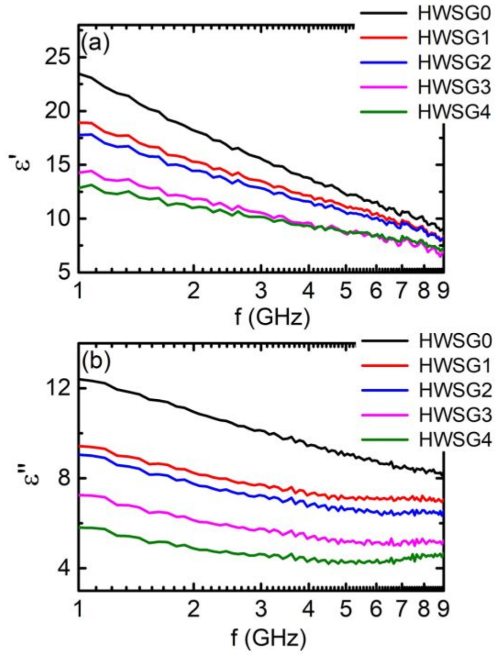

Figure S6. (a) Real and (b) Imaginary part of the measured dielectric permittivity for adulterated honey samples at various sugar concentrations.

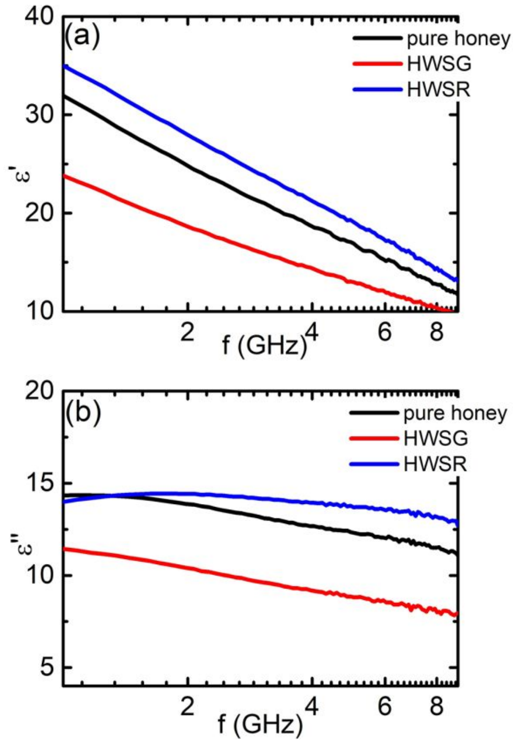

Figure S7. (a) Real and (b) Imaginary part of the measured dielectric permittivity for adulterated honey samples containing different adulterants at fixed concentrations.

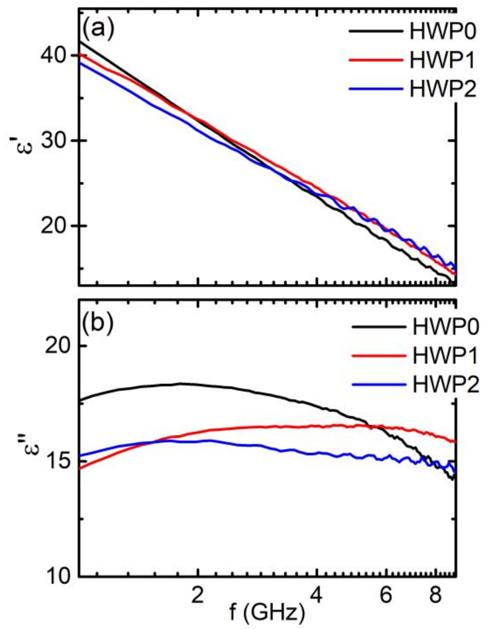

Figure S8. (a) Real and (b) Imaginary part of the measured dielectric permittivity for contaminated honey samples containing PET MPs.

Table S3: Raman peaks and corresponding assignments of adulterated honey solutions, at various sugar concentrations.

no.	sugar concentration					assignments (cm^{-1})
	0%	5%	10%	15%	20%	
1	352 (lowest)	352 (lowest)	358	362	362	357 ➔ δ(C–C–C) ring vibration in the pyranoid and furanoid forms of fructose ring from the sucrose spectrum [1]
2	836 (lowest)	842 (lowest)	836	832	832	836 ➔ v(C-C) in sucrose aqueous solution [2]
3	1064 (lowest)	1065	1067	1064	1064	1064 ➔ v(C-O) exo in sucrose aqueous solution [2]
4	1130 (lowest)	1127	1129	1130	1127	1130 ➔ δ(COH) in sucrose aqueous solution [2]<br>1127 ➔ sugar as adulterant [1]
5	1369 (lowest)	1373	1368	1367	1368	1366 ➔ w(CH_2) in sucrose aqueous solution [2]<br>1371 ➔ D-(+)-Trehalose (medium) [3]

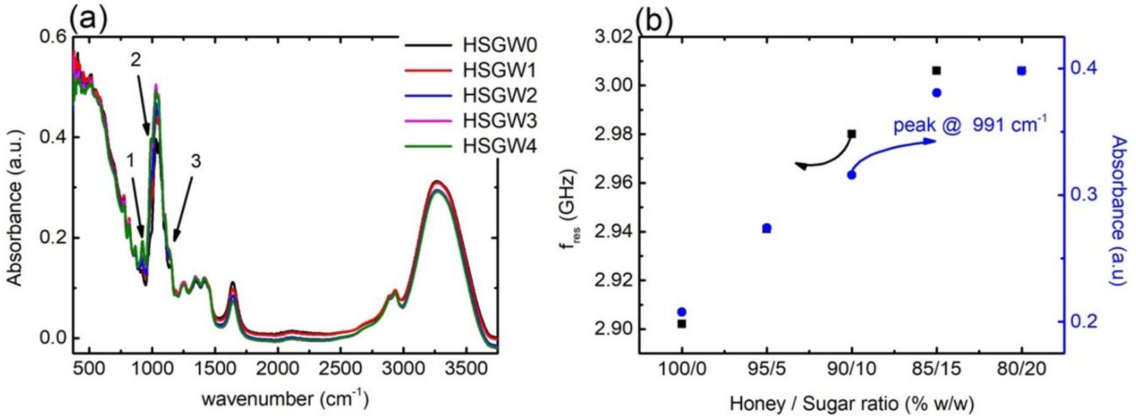

Figure S9. (a) FT-IR spectra for all honey samples adulterated with sugar at different percentages. Characteristic absorbance peaks indicating the sucrose presence due to adulteration. (b) Evolution of the absorbance peak No. 2 intensity at 991 cm^{-1}, with respect to honey / sugar mass ratio.

Table S4: Absorbance peaks and corresponding assignments of adulterated honey solutions, at various sugar concentrations.

no.	sugar concentration					ATR assignments (cm^{-1})
	0%	5%	10%	15%	20%	
1	918 (lowest)	918	920	922	924	921 ➔ presence of sucrose in adulterated honey [4]<br>922 ➔ sucrose [5]
2	991 (lowest)	991	991	991	989 (highest)	991 ➔ presence of sucrose in adulterated honey [4]
3	780 (lowest)	1146	1146	1142	1134	1148 ➔ υ(C-H) in carbohydrates or/and υ(C-O) in carbohydrates [6].

Table S5: Raman peaks and corresponding assignments of adulterated honey solutions containing different adulterating substances at fixed concentrations.

no.	adulterant			assignments (cm^{-1})
	pure honey	sugar	syrup	
1	538 (shoulder)	539	539	542 ➔ unknown for maple syrup [7].
2	-	742	744	744 ➔ sugars of honey [4].<br>742 ➔ vibrational bonds of carbohydrates, C-C, C-O stretching from maple syrup [7].
3	1065	1065	1065	1064 ➔ ν(C-O) exo in sucrose aqueous solution [8].

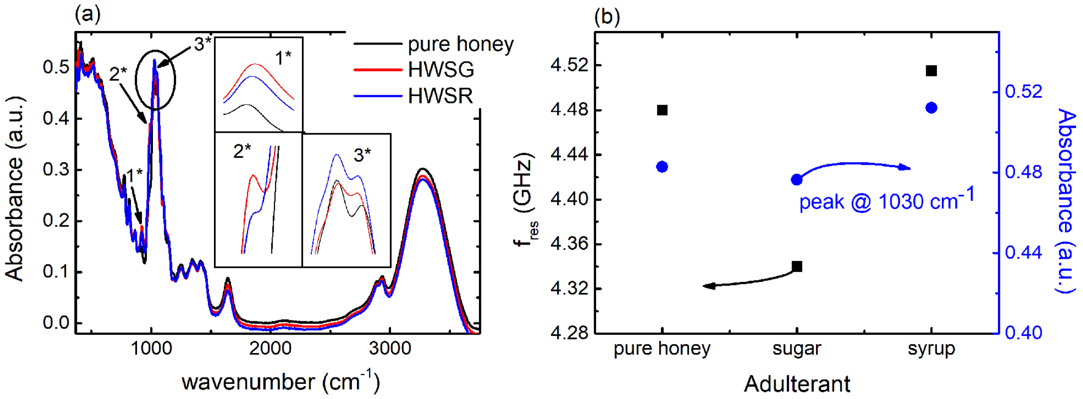

Figure S10. (a) FT-IR spectra for honey solutions containing different adulterating substances at a fixed concentration of 30% (w/w). (b) The evolution of resonance frequency and absorbance peak No. 3 at 1030 cm^{-1}, with respect to the adulterants used in this study.

Table S6: Absorbance peaks and corresponding assignments of adulterated honey solutions containing different adulterating substances.

no.	sample			ATR assignments (cm^{-1})
	pure honey	HWSG	HWSR	
1	918 (lowest)	922 (highest)	918	919 ➔ δ(C-H) [9]<br>921 ➔ presence of sucrose in adulterated honey [2]<br>922 ➔ sucrose [1]
2	–	991 (highest)	991 (shoulder)	991 ➔ presence of sucrose (in honey) [4]<br>992 ➔ sucrose [5]<br>991 ➔ stretching of C-O (C-OH groups) in syrup [7].
3	1030	1030	1030	1023➔ υ(C-O) in C-OH group or υ(C-C) in the carbohydrate structure [9].

References

(1) Ozbalci, B.; Boyaci, I. H.; Topcu, A.; Kadilar, C.; Tamer, U. Rapid Analysis of Sugars in Honey by Processing Raman Spectrum Using Chemometric Methods and Artificial Neural Networks. *Food Chem* **2013**, *136* (3–4), 1444–1452, DOI: 10.1016/j.foodchem.2012.09.064.

(2) Huzortey, A. A.; Arefi, A.; Anderson, B.; Khadem, H.; Sackey, S. S.; Mahmoodi-Khaledi, E.; Tavassoli, S. H. 532-Nm Laser-Excited Raman Spectroscopic Evaluation of Iranian Honey. *Food Anal. Methods* **2021**, *15*, 772–782, DOI: 10.1007/s12161-021-02164-4.

(3) De Gelder, J.; De Gussem, K.; Vandenabeele, P.; Moens, L. Reference Database of Raman Spectra of Biological Molecules. *J. Raman Spectrosc.* **2007**, *38* (9), 1133–1147, DOI: 10.1002/jrs.1734.

(4) Sotiropoulou, N. S.; Xagoraris, M.; Revelou, P. K.; Kaparakou, E.; Kanakis, C.; Pappas, C.; Tarantilis, P. The Use of SPME-GC-MS IR and Raman Techniques for Botanical and Geographical Authentication and Detection of Adulteration of Honey. *Foods* **2021**, *10* (7), DOI: 10.3390/foods10071671.

(5) Landari, H.; Roudjane, M.; Messaddeq, Y.; Miled, A. Pseudo-Continuous Flow FTIR System for Glucose, Fructose and Sucrose Identification in Mid-IR Range. *Micromachines (Basel)* **2018**, *9* (10), DOI: 10.3390/mi9100517.

(6) Antonova, O.; Calvo, J.; Seifert, A. Rapid Detection of Thermal Treatment of Honey by Chemometrics-Assisted FTIR Spectroscopy. *Foods* **2021**, *10* (11), DOI: 10.3390/foods10112892.

(7) Paradkar, M. M.; Sakhamuri, S.; Irudayaraj, J. Comparison of FTIR, FT-Raman, and NIR Spectroscopy in a Maple Syrup Adulteration Study. *J. Food Sci.* **2002**, *67* (6), 2009–2015, DOI: 10.1111/j.1365-2621.2002.tb09493.x.

(8) Tahir, H. E.; Xiaobo, Z.; Zhihua, L.; Jiyong, S.; Zhai, X.; Wang, S.; Mariod, A. A. Rapid Prediction of Phenolic Compounds and Antioxidant Activity of Sudanese Honey Using Raman and Fourier Transform Infrared (FT-IR) Spectroscopy. *Food Chem* **2017**, *226*, 202–211, DOI: 10.1016/j.foodchem.2017.01.024.

(9) Recklies, K.; Peukert, C.; Kölling-Speer, I.; Speer, K. Differentiation of Honeydew Honeys from Blossom Honeys and According to Their Botanical Origin by Electrical Conductivity and Phenolic and Sugar Spectra. *J. Agric. Food Chem.* **2021**, *69* (4), 1329–1347, DOI: 10.1021/acs.jafc.0c05311.
